# Soluble PD-L1 and Serum Vascular Endothelial Growth Factor-B May Independently Predict Prognosis in Patients with Advanced Non-Small Cell Lung Cancer Treated with Pembrolizumab

**DOI:** 10.3390/cancers17030421

**Published:** 2025-01-27

**Authors:** Eleni Kokkotou, Dimitra Grapsa, Anna Papadopoulou, Stylianos Gaitanakis, Petros Bakakos, Garyfallia Poulakou, Paraskevi Moutsatsou, Konstantinos Syrigos

**Affiliations:** 1 Oncology Unit, 3rd Department of Internal Medicine, Medical School, National and Kapodistrian University of Athens, 11527 Athens, Greece; dimgrap@yahoo.gr (D.G.); sgaitanakis@yahoo.com (S.G.); gpoulakou@gmail.com (G.P.); ksyrigos@med.uoa.gr (K.S.); 2 Laboratory of Clinical Biochemistry, “Attikon” University General Hospital, Medical School, National and Kapodistrian University of Athens, 11527 Athens, Greece; a_papado@yahoo.com (A.P.); pmoutsatsou@med.uoa.gr (P.M.); 3 1st Respiratory Medicine Department, Medical School, National and Kapodistrian University of Athens, 11527 Athens, Greece; petros44@hotmail.com

**Keywords:** immune checkpoint inhibitors, non-small cell lung cancer, sPD-1, sPD-L1, VEGF-A, VEGF-B

## Abstract

**Background:** Previous preclinical data have shown that the dynamic cross-talk between abnormal tumor vasculature and immune cell factors in the tumor microenvironment may exert a critical role in the progression and treatment resistance of non-small cell lung cancer (NSCLC). In the clinical setting, a variety of blood-based angiogenesis- and immune-related factors are being increasingly investigated as potential biomarkers of prognosis or treatment response in immunotherapy-treated NSCLC. We herein aimed to evaluate the clinical relevance of the peripheral blood levels of vascular endothelial growth factor-A and -B (VEGF-A and VEGF-B, respectively), soluble programmed cell death-1 (sPD-1), and programmed cell death-ligand 1 (sPD-L1) in patients with advanced NSCLC treated with immune checkpoint inhibitors (ICIs). **Methods:** Consecutive patients with advanced-stage, non-oncogene-addicted NSCLC, eligible to receive ICIs at the Oncology Unit of Sotiria Athens General Hospital, were prospectively recruited. A group of sex- and age-matched healthy controls was also enrolled for the evaluation of the potential diagnostic significance of the examined biomarkers. Serum levels of all biomarkers were measured using ELISA, both before and after treatment, and were correlated with standard clinicopathological features of patients, treatment response, progression-free survival (PFS), and overall survival (OS). **Results:** A total of 55 patients and 16 healthy controls were included in the final analysis. The mean age of patients and controls was 66.5 years (SD = 8.0 years) and 65.4 years (SD = 9.1 years), respectively. The majority of patients (65.5%) received pembrolizumab in combination with chemotherapy, while the remaining patients received pembrolizumab monotherapy. ROC curve analysis showed that VEGFB and sPD-1 were the only markers with a significant diagnostic value. Higher pre-treatment values of sPD-L1 (HR = 1.68; *p* = 0.040) and sPD-1 (HR = 10.96; *p* = 0.037) as well as higher post-treatment values of VEGF-B (HR = 2.99; *p* = 0.049) were all significantly associated with a reduced OS in univariate Cox regression analysis. The adverse prognostic significance of higher pre-treatment values of sPD-L1 (HR = 2.10; *p* = 0.014) and higher post-treatment values of VEGFB (HR = 3.37; *p* = 0.032) was further confirmed in multivariate analysis. **Conclusions:** Our study results suggest that serum levels of sPD-L1 and VEGF-B may independently predict prognosis in ICI-treated advanced-stage NSCLC.

## Simple Summary

In this prospective cohort study, the prognostic and predictive value of baseline and post-treatment levels of serum VEGF-A, VEGF-B, sPD-1, and sPD-L1 were investigated in 55 advanced NSCLC patients treated with immune checkpoint inhibitors (ICIs). Higher pretreatment sPD-L1 and posttreatment VEGF-B levels were found to independently predict worse overall survival. VEGF-A and sPD-1 failed to show a significant correlation with prognosis. None of the biomarkers was associated with treatment response. ICI-related toxicity was an independent predictor of response. VEGF-B and sPD-1 showed potential as diagnostic biomarkers, with significantly decreased levels in NSCLC patients compared to healthy controls.

## 1. Introduction

The advent of immune checkpoint inhibitors (ICIs) has revolutionized the treatment landscape in advanced non-small cell lung cancer (NSCLC), particularly in the subset of patients with non-oncogene-addicted disease, where ICIs, with or without chemotherapy, are the recommended first-line therapy [1]. Previous trial data have shown that first-line monotherapy with single-agent ICI in this setting may significantly prolong survival as compared to platinum doublet chemotherapy, particularly among patients with high programmed death-ligand 1 (PD-L1) expression, achieving as of yet unprecedented 5-year survival rates in this selected subgroup [2,3,4,5]. Most, but not all, observational studies seem to confirm these results in real-world practice as well, but the maximum benefit is again observed among patients resembling the selected populations of the above pivotal trials with regard to disease stage, performance status, and PD-L1 status, while older or more fragile patients often show significantly worse outcomes [6,7,8,9]. Evidently, not all patients derive a significant benefit from ICIs, while those who do benefit are at risk of developing autoimmune inflammatory responses known as immune-related adverse events (irAEs), which can be of variable severity, ranging from mild to life-threatening, and can affect almost any organ with a predilection for the endocrine system; furthermore, secondary resistance following the initial response ensues eventually, throughout the course of treatment, in the majority of cases [10,11,12,13].

The binding of PD-L1, a membrane protein primarily expressed on tumor cells, to its membrane-bound receptor PD-1, which is mainly expressed on activated T lymphocytes and other immune cells, is a major and well-known immune checkpoint pathway regulating the interaction between the immune system and cancer cells in the tumor microenvironment [14]. Immunohistochemical PD-L1 expression, routinely assessed on histological samples as tumor proportion score (TPS), is the most widely used predictor of ICI response until now, but its real-world applications are often limited by significant challenges, mainly including the need for invasive sampling methods, its heterogeneous expression in tumor tissue, reflecting the intratumoral heterogeneity of NSCLC, as well as the suboptimal standardization of the diagnostic assays employed and the variability of the scoring systems and cut-off values used [11,15,16]. Tumor mutational burden (TMB), an index reflecting the number of mutations per megabase (mut/Mb) in the tumor cell genome, and microsatellite instability (MSI), a condition of genetic hypermutability caused by deficiency of DNA mismatch repair (MMR) activity, are additional tissue-based biomarkers widely applied for prediction of ICI response, either independently or in combination with PD-L1 TPS, often providing stronger predictive information as compared to PD-L1 status alone, but failing as well to consistently provide clinically relevant and reproducible results or to accurately reflect the temporal and spatial heterogeneity of advanced NSCLC [16,17,18,19]. Therefore, there remains an unmet need to identify novel, ideally blood-based, predictive biomarkers in immunotherapy-treated NSCLC.

Abnormal tumor vasculature may interact with immune cells in the tumor microenvironment in a complex interplay, ultimately leading to immune suppression, which in turn may further drive angiogenesis, “in a vicious cycle of impaired immune activation”, as described by Rahma and Hodi [20]. VEGF-A, a protein with potent proangiogenic activity, may enhance immune system suppression in the tumor microenvironment (TME), as previously reported; more specifically, VEGF-A produced by tumor cells may enhance the expression of PD-1 and other inhibitory checkpoint molecules involved in the inactivation of CD8+ T cells [21]. Dysregulation of the architecture and functionality of the tumor vasculature may also prevent the effective delivery of T cells and anticancer agents in the tumor area, thus facilitating treatment resistance [22]. On the other hand, antiangiogenic therapy can reverse this process, first by normalizing the tumor blood vessels thus increasing perfusion and intra-tumoral tissue oxygenation, and second by restoring the normal function of immune cell factors in the TME, ultimately sensitizing the tumor to immunotherapy [22,23,24]. In accordance with these preclinical data, there is increasing clinical evidence that the combination of ICIs with antiangiogenic agents may not only be tolerable but also efficacious in prolonging survival as compared to ICI monotherapy in advanced NSCLC, while a variety of angiogenesis biomarkers, such as the VEGF family, are being studied for their potential utility as predictors of prognosis and treatment response in immunotherapy-treated NSCLC [24,25,26,27].

The primary aim of our study was to investigate the prognostic and predictive value of baseline and post-treatment levels of a panel of angiogenesis and immune-related markers, including serum vascular endothelial growth factor-A (VEGF-A), vascular endothelial growth factor-B (VEGF-B), soluble programmed cell death-1 (sPD-1), and programmed cell death-ligand 1 (sPD-L1) in patients with advanced NSCLC treated with ICIs. Secondary aims included the investigation of any potential correlations between the above candidate markers and the remaining clinicopathological features studied. Identification of robust blood-based biomarkers with the ability to accurately predict prognosis and/or treatment response throughout the course of disease and treatment may enhance the accuracy of prognostication and risk stratification and provide clinically relevant data for the designation of personalized treatment strategies with the ultimate aim to increase treatment efficacy while reducing the risk of unnecessary treatment-related toxicity in each individual patient.

## 2. Materials and Methods

### 2.1. Patient Population and Study Design

Consecutive patients with advanced NSCLC who were eligible to receive immunotherapy (as monotherapy or in combination with chemotherapy) at the Oncology Unit of Sotiria Athens General Hospital were prospectively enrolled. A group of sex- and age-matched healthy controls (n = 16) was recruited as well for determination of the optimal cut-off point between normal and increased serum levels of VEGF-A, VEGF-B, sPD-1, and sPD-L1. Controls were selected among patients referred to the 3rd Internal Medicine Department of our hospital and who were found to be free of any significant abnormality after diagnostic testing. The study protocol was approved by the ethics committee of our institution (approval number: 25244/25-10-19), and written informed consent was obtained from all participants prior to recruitment.

Inclusion criteria were defined as follows: written informed consent, age > 18 years, histologically or cytologically confirmed diagnosis of NSCLC, advanced disease stage (IIIB to IV), and eligibility for treatment with ICIs (non-oncogene addicted disease). Patients with operable NSCLC or severe comorbidities, significantly limiting life expectancy (e.g., end-stage cardiac, renal, or liver failure), or with other prior or concomitant malignancies were excluded from the study. Tumors were classified using the latest World Health Organization (WHO) histological classification. Staging was done according to the eighth edition of the International Association for Lung Cancer (IASLC) TNM classification system [28]. Standard staging procedures were used, including a complete history and physical examination, blood tests, computed tomography (CT) of the chest and abdomen, positron emission tomography (PET) scan, and a computed tomography (CT) scan (PET/CT scan) and CT or magnetic resonance imaging of the brain; bone involvement was documented by PET/CT scan or bone scintigraphy.

All patients were treated with ICIs, with or without chemotherapy; patients with non-squamous NSCLC received carboplatin/pemetrexed, while those with squamous cell lung cancer received carboplatin/paclitaxel or carboplatin/nab-paclitaxel, as determined by the treating physician in each individual case. Follow-up evaluations (clinical examination, CT scan, and routine laboratory investigations) were carried out at 3-month intervals. Treatment response was assessed after the completion of 3 treatment cycles using Response Evaluation Criteria in Solid Tumors (RECIST, version 1.1) and was classified as complete response (CR), partial response (PR), stable disease (SD), or progressive disease (PD). Progression-free survival (PFS) was calculated from the start date of the first treatment cycle to the time of first documentation of PD or until death by any cause. Overall survival (OS) was defined as the time from diagnosis to the date of death by any cause.

### 2.2. Sample Collection

Peripheral venous blood samples (for measuring serum levels of VEGF-A, VEGF-B, sPD-1, and sPD-L1) were collected from patients at the following time points: a) at baseline/before treatment (on the first day of the first cycle of immunotherapy treatment, before administering the therapeutic agent) and b) after treatment (at the time of treatment response evaluation). All samples were allowed to coagulate at room temperature for 30 min. The serum was separated by 2000× *g* for 10 min and stored at −20 °C until used for the ELISA measurements.

The levels of all biomarkers evaluated (pretreatment and posttreatment levels and the change between the two measurements) were correlated with standard clinicopathological features of patients, including age, sex, smoking history, disease stage, ECOG PS at diagnosis, histological type of tumor, immunohistochemical expression of PD-L1 in the primary tumor, type of treatment (monotherapy vs. combo), treatment-related toxicity (irAEs), treatment response, progression-free survival (PFS), and overall survival (OS).

### 2.3. ELISA Measurements

sPD-1, sPD-L1, VEGF-A, and VEGF-B serum levels were measured in all samples in duplicate using a quantitative sandwich enzyme immunoassay technique.

More specifically, sPD-1 was measured using the Human PD-1 sandwich ELISA Kit (ProteintechR KE00075) with intra-assay and inter-assay coefficients of variation (CV) less than 3.7% and 5.8%, respectively. The assay detection range was 125–8000 pg/mL. The minimum detectable dose of human PD1 is 43.0 pg/mL.

sPD-L1 was measured using the Human PD-L1 sandwich ELISA Kit (ProteintechR KE00074) with intra-assay and inter-assay coefficients of variation (CV) less than 7.8% and 7.0%, respectively. The assay detection range was 0.156–10 ng/mL. The minimum detectable dose of human PD1 is 0.04 ng/mL.

VEGF-A was measured using the Human VEGF-A ELISA Kit (InVitrogen BMS277-2) with minimum intra-assay and inter-assay coefficients of variation (CV) less than 11.7% and 8.9%, respectively. The detection range was 15.6–1000 pg/mL. The limit of detection was 7.9 pg/mL.

VEGF-B was measured using the Human VEGF-B ELISA Kit (InVitrogen EH481RB) with intra-assay and inter-assay coefficients of variation (CV) less than 10% and CV% < 12%, respectively. The detection range was 0.41–100 ng/mL. The minimum detectable dose of human VEGF-B was 0.4 ng/mL.

### 2.4. Statistical Analysis

Quantitative variables were expressed as mean (Standard Deviation) or median (interquartile range). Categorical variables were expressed as absolute and relative frequencies. For the comparison of proportions, chi-square and Fisher’s exact tests were used. Independent samples of Student’s *t*-tests were used for the comparison of age between patients and healthy participants. The Wilcoxon signed-rank test was used for the comparison of pre- and post-treatment biomarker values, while the Spearman correlation coefficient (rho) was used to measure the strength of correlation between two quantitative variables. ROC curves (Receiver Operating Characteristic curves) were used in order to estimate the diagnostic ability of biomarkers. Sensitivity, specificity, and negative and positive prognostic values were calculated for the determination of the optimal cut-offs. The area under the curve (AUC) was also calculated. The prognostic value of each biomarker was first assessed by univariate Cox regression analysis. Continuous variables were kept continuous in Cox regression analysis -were not dichotomized- to avoid loss of power and residual confounding, as previously described [29,30]. Variables that showed significant association with the outcome were included in the multivariate Cox proportional-hazard model in a stepwise method in order to determine the independent predictors for survival. The assumption of proportional hazards was evaluated by testing for interaction with a continuous time variable. Hazard ratios (HR) with 95% confidence intervals (95% CI) were computed from the Cox regression analyses. Kaplan–Meier survival estimates for survival were graphed over the follow-up period. All reported p-values are two-tailed. Statistical significance was set at *p* < 0.05, and analyses were conducted using SPSS statistical software (version 26.0).

## 3. Results

### 3.1. Clinicopathological Features of Patients

**Figure 1:**
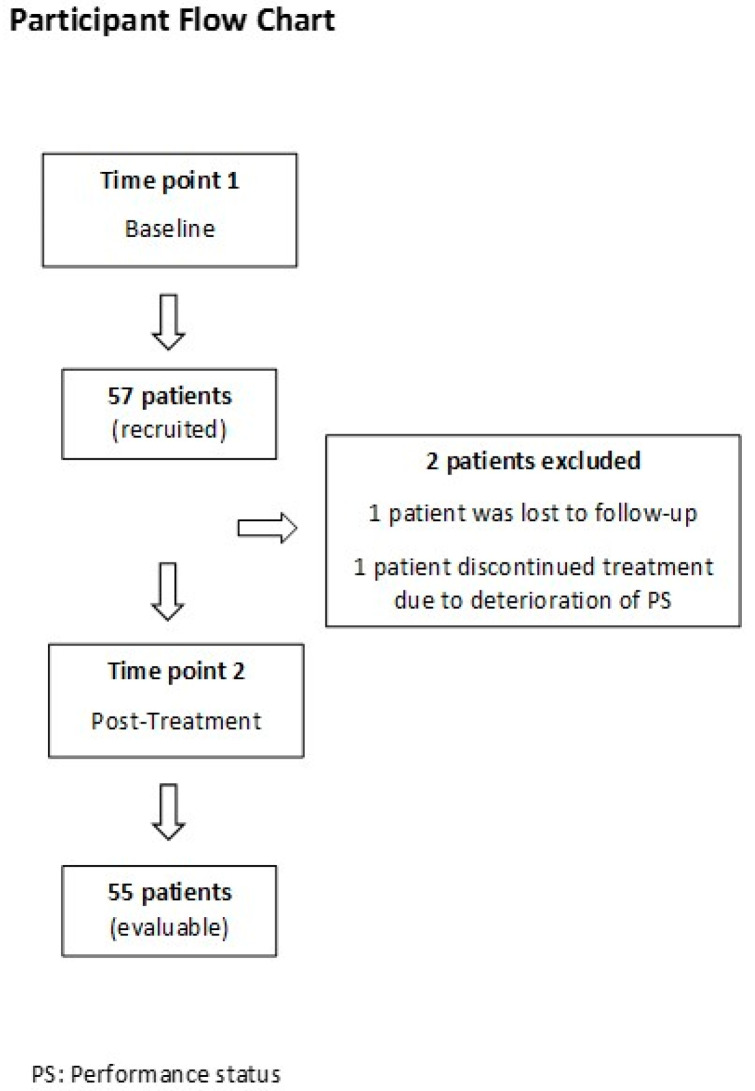
Participant flow chart.

A total of 55 patients and 16 healthy controls were enrolled and included for analysis in our study. The period of data collection was from January 2020 to July 2022. Only patients with both baseline and post-treatment samples available were included in the final analysis. Two patients who were initially recruited were eventually excluded from the study after baseline evaluation; one patient was lost to follow-up, and the second patient discontinued treatment due to deterioration of PS. The flow of participants throughout the study is depicted in Figure 1. The follow-up period ranged from 3 to 31 months (mean: 18.1 months).

The clinicopathological characteristics of all study participants are presented in Table 1. The mean age of patients and controls was 66.5 years (SD = 8.0 years) and 65.4 years (SD = 9.1 years), respectively. Also, the majority of both patients and healthy participants were males (69.1% and 56.3%, respectively). Patients and controls had similar age (*p* = 0.650) and gender (*p* = 0.339) distribution.

The histological type of tumor was adenocarcinoma In 35 cases (35/55, 63.6%), squamous cell carcinoma in 18 cases (18/55, 32.7%), adenosquamous carcinoma in 1 case (1/55, 1.8%), and NOS in 1 case as well (1/55, 1.8%). Most patients (51/55, 92.7%) had disease stage IV, ECOG PS 1 (34/55, 61.8%), and received first-line treatment (44/55, 84.6%). Pembrolizumab and chemotherapy combination was administered in 36/55 cases (65.5%), while the remaining patients (19/55, 34.5%) received pembrolizumab monotherapy. Disease progression was observed in 35.2% (19/55) and treatment-related toxicity (irAEs) in 40.0% (22/55) of patients.

### 3.2. Levels of Biomarkers and Their Diagnostic Accuracy

Pre- and post-treatment levels of the examined biomarkers are summarized in Table 2. Significant changes (between pre- and post-treatment values) were found only in VEGFB (*p* = 0.028) and sPD-1 (*p* < 0.001). More specifically, VEGFB decreased significantly after treatment, while sPD-1 increased significantly.

The diagnostic ability of the biomarkers evaluated was examined via ROC analysis (Table 3).

**Figure 2:**
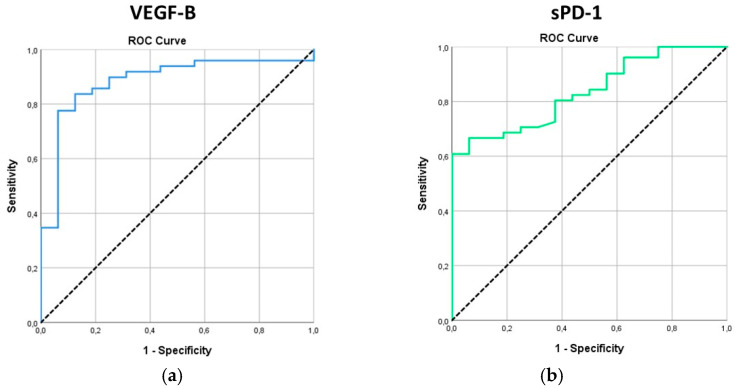
ROC curves for (**a**) VEGF-B and (**b**) sPD-1.

VEGFB and sPD-1 were the only markers showing a significant diagnostic value (Figure 2a,b).

The optimal cut-off for VEGFB was ≤10.94 pg/mL, with a sensitivity of 83.7% and specificity of 87.5%. Also, 83.7% (n = 41) of the patients had VEGFB ≤ 10.94, while the corresponding percentage for controls was 12.5% (n = 2) (*p* < 0.001). The optimal cut-off for sPD-1 was ≤34.54 pg/mL, with a sensitivity of 80.4% and a specificity of 62.5%. Also, 80.4% (n = 41) of patients had sPD-1 ≤ 34.54, while the corresponding percentage for controls was 37.5% (n = 6) (*p* = 0.003).

### 3.3. Associations Between Biomarkers and Clinicopathological Features

Higher posttreatment VEGFA and sPD-L1 levels were associated with 2nd or 3rd line therapy. Higher posttreatment sPD-L1 levels were also correlated with the administration of pembrolizumab monotherapy.

Spearman’s correlation analysis showed a significant correlation between the following variables: (a) higher pack-years and greater change in sPD-L1, (b) higher tumor PD-L1 (TPS) and higher post-treatment sPD-L1 and sPD-1, (c) higher tumor PD-L1 and greater change in sPD-1. Spearman’s correlation analysis also revealed various correlations between the study biomarkers (presented in Table 4).

### 3.4. Correlation with Treatment Response and Survival Analysis

The mean time to disease progression was 26.9 months (SE = 2.5 months). Only irAEs were found to significantly correlate with treatment response, both in univariate and in multivariate analysis; more specifically, patients who developed irAEs had a reduction of hazard for disease progression by 87% as compared to patients with no toxicity (HR = 0.13; *p* = 0.006).

**Figure 3:**
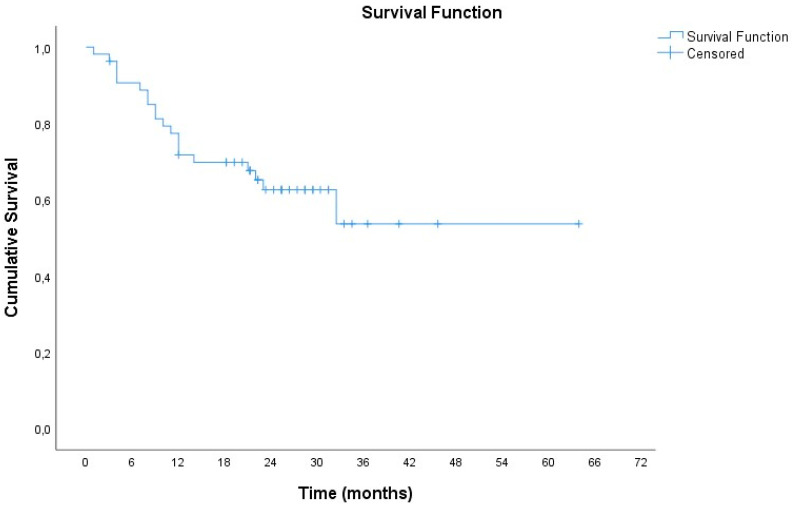
Kaplan–Meier curve for survival of patients.

During follow-up, 38.2% of patients (n = 21) died, and the mean survival time was 41.2 months (SE = 4.3 months) (Figure 3).

Univariate Cox regression analysis showed that higher pre-treatment values of sPD-L1 (HR = 1.68; *p* = 0.040) and sPD-1 (HR = 10.96; *p* = 0.037) were significantly associated with greater hazard (Table 5).

Similarly, higher post-treatment values of VEGF-B (HR = 2.99; *p* = 0.049) were significantly associated with greater hazard. Multivariate Cox regression analysis results are presented in Table 6.

Higher pre-treatment values of sPD-L1 (HR = 2.10; *p* = 0.014) and higher post-treatment values of VEGFB (HR = 3.37; *p* = 0.032) were significantly associated with greater hazard.

## 4. Discussion

In the present study, higher serum levels of pretreatment sPD-L1 and posttreatment VEGF-B were found to independently predict a worse OS in ICI-treated advanced-stage NSCLC. The remaining biomarkers evaluated, i.e., VEGF-A and sPD-1, failed to show any statistically significant correlation with prognosis, while none of the blood-based biomarkers included in our panel was found to be significantly associated with treatment response. An independent predictive value was revealed for ICI-related toxicity only, among all parameters studied, thus confirming its well-established role as a clinical predictor of response to ICI in this setting [31].

Given the intrinsic limitations of tissue biomarkers, such as PD-L1 TPS, especially for the purpose of longitudinal real-time monitoring of patients with advanced NSCLC, there is an ongoing quest for the identification of blood-based predictors, enabling repeat evaluations throughout the disease and treatment course, without the need for invasive sampling methods. Both PD-1 and PD-L1 checkpoint molecules can be detected not only on tissue samples (as membrane-bound PD-1 and PD-L1) but also in the peripheral blood as well, in the form of soluble proteins (sPD-1 and sPD-L1), thus enabling convenient monitoring of their levels at any time point needed. Most previous studies investigating baseline sPD-L1 in ICI-treated NSCLC seem to generally concur that it may represent a useful prognostic and predictive biomarker in this setting. A recent meta-analysis concluded that high sPD-L1 may predict a worse OS and PFS in lung cancer patients treated with ICIs [32], confirming the results of three previous meta-analyses [33,34,35] and in agreement with the results presented herein.

Previous data on the clinical relevance of sPD-1 in ICI-treated NSCLC are much more limited (as compared to PD-L1) but seem to largely concur that higher posttreatment sPD-1 levels may represent a marker of improved survival. As reported by Himuro et al. [36], increased sPD-L1 levels at baseline were significantly correlated with worse PFS and OS in NSCLC patients receiving ICI monotherapy but not in those receiving ICIs-chemotherapy combinations, while higher posttreatment sPD-1 and PD-L1 levels were predictive of improved and worse OS, respectively, suggesting, as emphasized by the authors, that both pretreatment sPD-L1, as well as posttreatment sPD-1 and sPD-L1, may represent useful prognostic biomarkers in this setting. In another study, posttreatment sPD-1 levels were again correlated with improved OS in the ICI monotherapy subgroup of NSCLC patients [37]. Interestingly, Ohkuma et al. [38] suggested a potential involvement of sPD-1 in primary resistance to anti-PD-1 ICIs in patients with various solid tumors, including NSCLC, and that early changes of this marker during the course of treatment may help identify patients least likely to respond. Furthermore, a composite sPD-L1/sPD-1 biomarker for the prediction of ICI efficacy has also been proposed, based on the observed independent correlation of baseline positivity for both markers with a worse PFS [39]. In our study, we failed to demonstrate any correlation between sPD-1 levels and treatment response or survival, but this may be due to the inclusion of patients receiving both ICI monotherapy and ICI-chemotherapy combination.

The pathophysiological mechanisms underlying the above clinical observations remain to be fully elucidated. As previously hypothesized, the soluble forms of PD-1 and PD-L1 may retain a binding ability similar to their membrane-bound counterparts as well as the capacity to exert significant biological actions; sPD-L1 may bind to sPD-1 on T cells, thus disrupting their activation and enhancing the immune escape of tumor cells, while, in contrast, sPD-1 may bind to and potentially inhibit membranous-bound PD-L1, thus leading to restoration of the antitumor T-cell function [36,40,41]. Therefore, sPD-L1 and sPD-1 seem to exert opposite effects on the tumor microenvironment, as a promoter and repressor of tumor growth, respectively. Notably, an additional obstacle in the delineation of the exact clinical relevance of sPD-1 and sPD-L1 in NSCLC is the existing ambiguity regarding the exact source of these secreted proteins in the peripheral circulation, particularly with regard to sPD-L1. sPD-1, encoded by the PDCD1 gene, is thought to be primarily generated by alternative mRNA splicing; sPD-L1 is also thought to be produced by alternative splicing of the PD-L1 mRNA as well as by proteolytic cleavage of the extracellular fraction of membrane-bound PD-L1 expressed on tumor cells or mature dendritic cells; some sPD-L1 isoforms are also believed to originate from proteolysis of PD-L1 expressed on the surface of secreted cellular exosomes [42,43,44]. Recently, Teramoto et al. [45] reported that sPD-L1 in the peripheral blood of patients with operable NSCLC can be generated not only from PD-L1-expressing tumor cells but also from PD-L1-positive tumor-associated macrophages (TAMs) and that prognosis among patients with elevated baseline sPD-L1 levels may depend on the primary source of sPD-L1 in the circulation. These intriguing observations further highlight the complexity of the pathophysiological mechanisms by which sPD-L1 and sPD-1 exert their biological actions in NSCLC and guide the focus of future translational research in this field.

Despite the well-established interplay between angiogenesis and immune cell factors in the tumor microenvironment [20,23,24], exerting a critical role in the progression of NSCLC and its response to immunotherapy, research on the potential prognostic and predictive significance of peripheral blood levels of angiogenesis markers in ICI-treated NSCLC, especially with regard to VEGF-B, is sparse. In a previous study combining preclinical and clinical research, VEGF-B was shown to promote metastasis in human and mouse tumor models through remodeling of tumor microvasculature, in a process seemingly independent of VEGF-A (also known as VEGF), and despite parallel suppression of primary tumor growth; furthermore, high VEGF-B tumor tissue expression was shown to correlate with worse survival in two separate cohorts of patients with squamous cell lung cancer and melanoma, respectively, suggesting that VEGF-B may adversely impact prognosis [46]. On the other hand, decreased tissue expression of VEGF-B (along with increased VEGF-A expression) was correlated with worse time to progression (TTP) and OS in resectable NSCLC in another study [47]. As reported by Lee et al. [48], VEGF-B can be pro- or anti-angiogenic depending on additional factors, such as the expression status and levels of the fibroblast growth factor 2 (FGF2) and Fibroblast growth factor receptor 1 (FGFR-1), two potent angiogenic molecules with the ability to act as a “switch” of VEGFB function; when FGF2/FGFR1 levels are high, VEGF-B may exhibit an anti-angiogenic function by inhibiting the FGF2/FGFR1 pathway, while in the absence of FGF2/FGFR1 expression or when their levels are low, VEGF-B may exert pro-angiogenic activity, enhancing blood vessel survival. Increased levels of posttreatment VEGF-B were shown to independently correlate with reduced OS in our study, thus suggesting that VEGF-B may represent an adverse prognostic indicator in ICI-treated NSCLC. To the best of our knowledge, there is no previous study evaluating the prognostic and predictive significance of serum VEGF-B levels in this setting. Therefore, additional investigations of this candidate biomarker are warranted to further support our preliminary observations.

Previous studies on the potential prognostic and predictive relevance of VEGF-A levels in the peripheral blood of patients with ICI-treated NSCLC are limited. Continuous decrease in plasma VEGF-A levels, from baseline to day 14 of treatment, was previously correlated with prolonged PFS in patients with advanced NSCLC receiving chemotherapy-ICI combination therapy, suggesting the potential predictive value of this marker [49]. In another study, lower baseline sPD-L1 and higher post-treatment VEGF levels were both independently associated with increased and reduced PFS, respectively, in NSCLC patients treated with PD-L1 inhibitors combined with anti-angiogenetic therapy [50]. Hu et al. [51] reported that increased VEGF levels at baseline were correlated with worse PFS in advanced non-small cell lung cancer treated with ICI, while Shibaki et al. [52] similarly observed a worse overall response rate to anti-PD-1 antibody treatment among fragile patients with advanced NSCLC and higher serum VEGF levels, thus reinforcing the hypothesis that increased VEGF levels may be predictive of reduced efficacy to these agents.

Although not our primary aim, in the present study we also found a potential value of VEGF-B and sPD-1 as diagnostic biomarkers. More specifically, VEGF-B and sPD-1 levels were both found to be significantly decreased in NSCLC patients as compared to healthy sex- and age-matched controls and to be able to discriminate between patients and controls with an optimal cut-off of 10.94 pg/mL and 34.54 pg/mL, respectively. These findings seem to be in contrast to some previous studies reporting increased sPD-1 levels in the serum or plasma of patients with advanced NSCLC as compared to healthy controls [53,54]. Peng et al. [53] investigated the clinical significance of sPD-1 and other soluble immune checkpoint markers, including sTIM-3, sCD137, sCD27, sLAG-3, sIDO, sPD-L2, sCD152, and sCD8 in NSCLC and reported increased serum levels of all examined biomarkers (including sPD-1) in patients with advanced-stage disease versus controls; nevertheless, sPD-1 was the only marker that failed to confirm its diagnostic value in subsequent ROC analysis while a higher diagnostic accuracy was reached when a combined detection assay of sTIM-3, sLAG-3, and sCD137 was performed [53]. On the other hand, our own current findings of decreased sPD-1 levels in advanced NSCLC as compared to controls concur with those recently reported by Gu et al. [55]. In the latter study, sPD-1 serum levels were found to be significantly reduced in NSCLC, and the authors hypothesized that this striking observation of a reduction instead of an increase in sPD-1 levels might be due to either reduced production of sPD-1 or to increased expression of its ligand PD-L1 [55].

The observed discrepancies among studies evaluating the diagnostic, prognostic, or predictive significance of sPD-1 and sPD-L1 in NSCLC may, at least partly, be explained by their significant heterogeneity with regard to several key pre-analytical and analytical methodological parameters, mainly including differences in the type of biological sample (plasma vs. serum) analyzed, the assays employed (i.e., use of different kits), and the thresholds used as cut-offs. As previously emphasized, a major obstacle in the implementation of sPD-L1 as a prognostic or predictive biomarker in routine practice is the absence of standardized methods of sPD-L1 measurement, with different ELISA kits and thresholds used by different research groups [41,56]. The latter parameter may be of considerable significance, given the lack of pre-established cut-off values for these candidate biomarkers and the use of different statistical analysis methods or variable types of cut-offs for prognostic purposes in the research setting (analysis of continuous variables without dichotomization, use of median values as cut-offs or use of cut-offs determined with ROC analysis). Standardization of all the above factors is, therefore, a prerequisite for the validation and subsequent implementation of these biomarkers, or any blood-based biomarker for that matter, in routine clinical practice. Furthermore, differences with regard to the sample size or the inclusion criteria of patients, the time points of sample collection, or the duration of follow-up represent additional significant sources of heterogeneity and may also lead to inconsistent results between studies.

Although strengthened by the prospective design of our study and the evaluation of all clinical aspects (diagnostic, prognostic, and predictive) of the examined biomarkers, our results need to be evaluated in the context of some limitations as well. First, our patient population sample was relatively small, limiting the statistical power of our analysis and the ability to perform statistical analysis in subgroups of patients stratified by type of therapy (pembrolizumab plus chemotherapy and pembrolizumab monotherapy subgroups). To minimize the latter limitation, the type of ICI treatment (pembrolizumab monotherapy vs. combo) was included as a variable in our study and found to be correlated with higher posttreatment sPD-L1 levels only, while failing to show a significant association with any other parameter, including, of course, prognosis and treatment response. An additional limitation of our study is the fact that patients receiving second-line treatment and beyond were also included, albeit as a minority subgroup (15.4%), thus reducing the homogeneity of our population but also better reflecting the characteristics of a real-world cohort. Finally, it must also be emphasized that our findings warrant additional confirmation in a validation cohort.

## 5. Conclusions

Our study results revealed an independent prognostic significance of pretreatment sPD-L1, thus confirming previous reports, but also highlighted serum VEGF-B, a biomarker relatively understudied as of yet, as a novel predictor of prognosis in ICI-treated advanced NSCLC. Undoubtedly, additional studies are needed to delineate the exact role of these candidate biomarkers, particularly VEGF-B, in treatment response and overall prognosis of patients. Optimization of ICI-based treatment planning in advanced NSCLC will, most likely, require the designation of a combination of markers instead of a single one, following their robust validation in large prospective series.

## Figures and Tables

**Table 1:** Demographics and clinicopathological features of patients and controls.

		n (%)
Patients (n = 55)		
***Gender***		
	Male	38 (69.1)
	Female	17 (30.9)
***Age (years), mean (SD)/median***		66.5 (8.0)/67
***Pack-Years, mean (SD)/median***		64.7 (27.6)/60
***ECOG Performance Status***		
	0	12 (21.8)
	1	34 (61.8)
	2	8 (14.5)
	3	1 (1.8)
***Type of treatment***		
	Pembrolizumab monotherapy	19(34.5)
	Pembrolizumab + chemotherapy	36 (65.5)
***Treatment line***		
	1	44 (84.6)
	2	7 (13.5)
	3	1 (1.9)
***Disease Stage***		
	ΙΙΙ	4 (7.3)
	IV	51 (92.7)
***Histological type of tumor***		
	Adenocarcinoma	35 (63.6)
	Squamous cell carcinoma	18 (32.7)
	Adenosquamous carcinoma	1 (1.8)
	NOS	1 (1.8)
***PD-L1, mean (SD)/median***		44.7 (36)/50
***Response to treatment***		
	Partial response	14 (25.9)
	Stable disease	21 (38.9)
	Disease progression	19 (35.2)
***Toxicity (irAEs)***		22 (40.0)
**Healthy controls (n = 16)**		
***Gender***		
	Male	9 (56.3)
	Female	7 (43.8)
***Age (years), mean (SD)/median***		65.4 (9.1)/66

**Table 2:** Pretreatment and posttreatment levels of the examined biomarkers.

	n	Mean (SD)	Median (IQR)
**Pre-treatment**			
VEGFA	51	504.86 (311.46)	433.05 (221.39–731.57)
VEGFB	49	77.22 (474.79)	4.97 (3.02–8.2)
sPD-L1	51	0.17 (0.09)	0.16 (0.09–0.25)
sPD-1	51	18.17 (17.52)	12.29 (3.75–29.17)
**Post-treatment**			
VEGFA	42	430.17 (286.33)	321.19 (214.66–650.97)
VEGFB	42	17.69 (31.66)	6.44 (4.13–16.8)
sPD-L1	43	0.2 (0.11)	0.16 (0.11–0.3)
sPD-1	42	56.22 (99.02)	40.31 (22.92–52.68)
**Change**			
VEGFA	40	−62.95 (218.99)	−19.26 (−148.18–94.36)
VEGFB	38	−78.62 (514.62)	1.74 (−1.18–3.83)
sPD-L1	41	0.02 (0.13)	0.01 (−0.06–0.09)
sPD-1	40	39.97 (102.04)	19.06 (6.73–39.07)

**Table 3:** Levels of biomarkers in patients and controls and their diagnostic ability, via ROC analysis.

	Patients		Healthy Sample									
	Mean (SD)	Median (IQR)	Mean (SD)	Median (IQR)	ROC	95% CI	*p*	Optimal Cut-Off	Sensitivity (%)	Specificity (%)	PPV (%)	NPV (%)
**VEGFA**	504.86 (311.46)	433.05 (221.39–731.57)	410.46 (222.32)	347.39 (256.17–552.22)	0.58	0.44–0.72	0.339	-	-	-	-	-
**VEGFB**	77.22 (474.79)	4.97 (3.02–8.2)	53.39 (39.45)	44.77 (16–100)	0.88	0.79–0.98	<0.001	≤10.94	83.7	87.5	95.3	63.6
**sPD-L1**	0.17 (0.09)	0.16 (0.09–0.25)	0.19 (0.1)	0.15 (0.12–0.21)	0.47	0.32–0.62	0.702	-	-	-	-	-
**sPD-1**	18.17 (17.52)	12.29 (3.75–29.17)	73.14 (119.28)	39.09 (25.17–63.39)	0.83	0.74–0.93	<0.001	≤34.54	80.4	62.5	87.2	50

Note. CI: Confidence Interval; PPV: Positive Prognostic value; NPV: Negative Prognostic Value.

**Table 4:** Spearman’s correlation analysis results for all studied biomarkers.

		Pretreatment			Posttreatment				Change			
		VEGFB	sPD-L1	sPD-1	VEGFA	VEGFB	sPD-L1	sPD-1	VEGFA	VEGFB	sPD-L1	sPD-1
**Pretreatment**												
**VEGFA**	rho	−0.04	0.19	−0.16	0.76	0.16	0.48	0.23	−0.37	0.31	0.25	0.35
	P	0.780	0.192	0.256	**<0.001**	0.330	**0.002**	0.160	**0.019**	0.061	0.109	**0.025**
**VEGFB**	rho	1.00	0.26	0.09	−0.04	0.45	0.17	0.00	0.08	−0.26	−0.04	−0.05
	P		0.074	0.540	0.814	**0.005**	0.298	0.999	0.628	0.118	0.786	0.743
**sPD-L1**	rho		1.00	0.07	0.19	0.05	0.25	−0.12	−0.17	−0.17	−0.54	−0.10
	P			0.622	0.234	0.747	0.109	0.477	0.308	0.316	**<0.001**	0.545
**sPD-1**	rho			1.00	−0.36	0.38	0.18	0.32	−0.33	0.29	0.10	−0.34
	P				**0.022**	**0.014**	0.262	**0.041**	**0.040**	0.081	0.522	**0.031**
**Posttreatment**												
**VEGFA**	rho				1.00	0.03	0.29	0.01	0.23	0.14	0.22	0.28
	P					0.851	0.067	0.974	0.155	0.393	0.172	0.085
**VEGFB**	rho					1.00	0.26	0.30	−0.10	0.55	0.19	0.08
	P						0.101	**0.050**	0.537	**<0.001**	0.231	0.627
**sPD-L1**	rho						1.00	0.43	−0.25	0.11	0.62	0.35
	P							**0.005**	0.119	0.502	**<0.001**	**0.028**
**sPD-1**	rho							1.00	−0.42	0.34	0.56	0.74
	P								**0.008**	**0.038**	**<0.001**	**<0.001**
**Change**												
**VEGFA**	rho								1.00	−0.20	−0.10	−0.20
	P									0.236	0.536	0.222
**VEGFB**	rho									1.00	0.26	0.21
	P										0.120	0.212
**sPD-L1**	rho										1.00	0.49
	P											**0.001**

No other statistically significant associations were observed between the biomarkers evaluated (pretreatment, post-treatment, and change between the two measurements) and the remaining clinicopathological features and treatment data of patients (sex, age, ECOG PS, disease stage, and treatment-related toxicity).

**Table 5:** Univariate Cox analysis results for survival, with biomarkers as independent variables.

	HR (95% CI) ^1^	*p*
**Pre-treatment**		
VEGFA	0.97 (0.83–1.13)	0.673
VEGFB	1.03 (0.97–1.10)	0.297
sPD-L1	1.68 (1.02–2.74)	**0.040**
sPD-1	10.96 (1.15–104.19)	**0.037**
**Post-treatment**		
VEGFA	1.02 (0.85–1.23)	0.828
VEGFB	2.99 (1.01–8.89)	**0.049**
sPD-L1	0.30 (0.00–28.71)	0.601
sPD-1	0.90 (0.45–1.85)	0.777
**Change**		
VEGFA	0.94 (0.74–1.20)	0.628
VEGFB	0.96 (0.90–1.03)	0.243
sPD-L1	0.02 (0.00–1.63)	0.079
sPD-1	0.82 (0.35–1.96)	0.659

^1^ Hazard Ratio (95% Confidence Interval).

**Table 6:** Multivariate Cox analysis results for survival in a stepwise method.

	HR (95% CI) ^1^	*p*
sPD-L1 pre-treatment	2.10 (1.16–3.80)	**0.014**
VEGFB post-treatment	3.37 (1.11–10.22)	**0.032**

^1^ Hazard Ratio (95% Confidence Interval).

## Data Availability

The original contributions presented in the study are included in the article.
